# Survival among patients with advanced renal cell carcinoma in the pretargeted versus targeted therapy eras

**DOI:** 10.1002/cam4.574

**Published:** 2015-12-08

**Authors:** Pengxiang Li, Yu‐Ning Wong, Katrina Armstrong, Naomi Haas, Prasun Subedi, Margaret Davis‐Cerone, Jalpa A. Doshi

**Affiliations:** ^1^ Division of General Internal MedicineUniversity of PennsylvaniaPhiladelphiaPennsylvania; ^2^ Leonard Davis Institute of Health EconomicsPhiladelphiaPennsylvania; ^3^ Fox Chase Cancer CenterPhiladelphiaPennsylvania; ^4^ Massachusetts General HospitalBostonMassachusetts; ^5^ Hospital of the University of PennsylvaniaPhiladelphiaPennsylvania; ^6^ Pfizer Inc.New YorkNew York

**Keywords:** Advanced renal cell carcinoma, metastatic kidney cancer, population‐based study, survival, targeted therapies

## Abstract

Between December 2005 and October 2009, FDA approved six targeted therapies shown to significantly extend survival for advanced renal cell carcinoma (RCC) patients in clinical trials. This study aimed to examine changes in survival between the pretargeted and targeted therapy periods in advanced RCC patients in a real‐world setting. Utilizing the 2000–2010 SEER Research files, a pre–post study design with a contemporaneous comparison group was employed to examine differences in survival outcomes for patients diagnosed with advanced RCC (study group) or advanced prostate cancer (comparison group, for whom no significant treatment innovations happened during this period) across the pretargeted therapy era (2000–2005) and the targeted therapy era (2006–2010). RCC patients diagnosed in the targeted therapy era (*N* = 6439) showed improved survival compared to those diagnosed in the pretargeted therapy era (*N* = 7231, hazard ratio (HR) for all‐cause death: 0.86, *P* < 0.01), while the change between the pre–post periods was not significant for advanced prostate cancer patients (HR: 0.97, *P *= 0.08). Advanced RCC patients had significantly larger improvements in overall survival compared to advanced prostate cancer patients (*z* = 4.31; *P* < 0.01). More detailed year‐to‐year analysis revealed greater survival improvements for RCC in the later years of the posttargeted period. Similar results were seen for cause‐specific survival. Subgroup analyses by nephrectomy status, age, and gender showed consistent findings. Patients diagnosed with advanced RCC during the targeted therapy era had better survival outcomes than those diagnosed during the pretargeted therapy era. Future studies should examine the real‐world survival improvements directly associated with targeted therapies.

## Introduction

Renal cell carcinoma (RCC) accounts for approximately 90–95% of all types of kidney cancers and 3% of adult malignancies in the United States 1. RCC often lacks early warning signs, and up to 30% of cases are diagnosed at an advanced or metastatic stage 2. The prognosis for metastatic RCC patients has been historically poor, with a 5‐year survival rate of lower than 10% 3. Prior to 2006, systemic treatment of advanced RCC was limited to the cytokines: interferon‐alfa and interleukin‐2. These drugs have demonstrated limited activity and have been associated with considerable toxicities 4.

Between December 2005 and October 2009, a total of six targeted agents—four that target angiogenesis and two mammalian rapamycin (mTOR)–targeted therapies—were approved for the treatment of advanced RCC by the U.S. Food and Drug Administration (FDA). A seventh targeted therapy was approved in January 2012. The rapid pace of drug development within a short time frame significantly altered the treatment paradigm for advanced RCC. These novel agents became the first line of therapy, offering patients multiple treatment options that have been shown to significantly extend survival in clinical trials 5, 6, 7. However, limited evidence exists regarding how these pharmaceutical innovations impact survival in real‐world settings.

A few U.S.‐based studies have examined improvements in survival among RCC patients in routine care after the introduction of these novel therapies 8, 9, 10, 11. With the exception of one study with unclear study methods 11, these studies reported statistically significant survival improvements among patients with advanced RCC. However, a key issue across all studies was the use of a pre–post only study design without a comparison group. Hence, their findings of improvements in survival in the targeted therapy era relative to the pretargeted therapy era could be confounded by contemporaneous trends (e.g., advances in imaging and supportive care). Furthermore, several methodological issues across these studies may have resulted in an underestimation of the pre–post change in survival. First, certain groups of patients who would not be considered candidates for the targeted therapies were not excluded in some studies 9. Second, patients diagnosed in the pretargeted therapy era were not censored before the start of the targeted therapy era; as a result, use of targeted therapies by these patients in later years likely contributed to improvements in their survival 8, 9, 10, 11. Third, prior studies classified patients diagnosed between 2004 9 and 2005 8, 10 into the post period (i.e., targeted therapy era) group even though the first targeted therapy was not FDA‐approved until December 2005. Fourth, studies did not utilize data beyond 2009, which did not permit capture of the effects of the availability of three new targeted therapies that became available that year.

We sought to address these limitations by examining population‐based changes in survival outcomes from the pretargeted to the targeted therapy era in advanced RCC patients relative to a contemporaneous comparison group (advanced prostate cancer patients) using data from 2000 to 2010. To provide additional insights into whether the availability of multiple new agents may have led to survival gains on an incremental basis, we also examined changes in survival for each year of the posttargeted period compared to the preperiod among RCC patients.

## Methods

### Study design and data source

We used a pre–post study design with a contemporaneous comparison group to examine differences in survival outcomes for patients first diagnosed with advanced cancer across two periods: “pretargeted (2000–2005),” in which only cytokine therapy was available, and “targeted (2006–2010),” during which angiogenesis therapies and m‐TOR therapies also became available upon FDA approval. Data were drawn from the 2000–2010 Surveillance, Epidemiology, and End Results (SEER) research database, which includes cancer registry data from 18 sites representing 15 states in the United States and captures cancer incidence for approximately 28% of the U.S. population. SEER includes information on cancer site, histology, stage, treatment, and survival 12.

#### Study group

**Figure A1:**
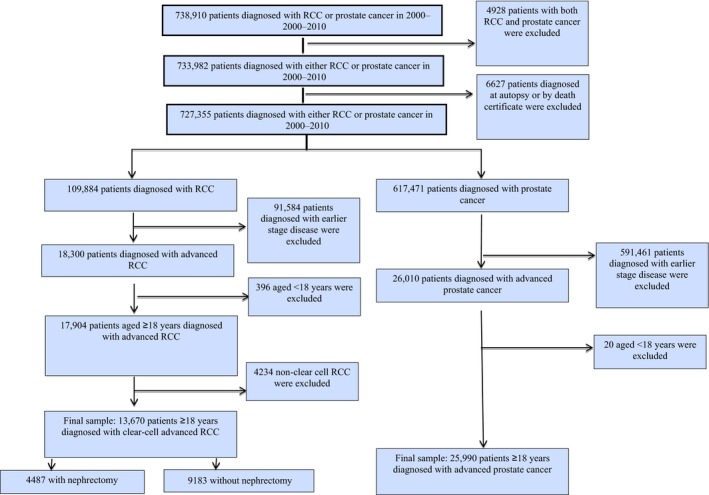
Sample selection diagram.

We sought to capture adults aged ≥18 years diagnosed with metastatic RCC (ICD‐O‐2/3 site codes C64.9) with clear cell histology (i.e., who would be candidates for targeted therapy) in our sample. Although the 2004–2009 SEER dataset contains variables that enable easy identification of individuals who have been diagnosed with metastatic disease, data from 2000–2003 are not quite as detailed. Thus, we utilized the SEER variable on summary and historical stage that was consistently available in each year of our 2000–2010 study time frame (SEER Cancer Stage Variable: hist_ssg_2000=7: Distant site(s)/node(s) involved). To assess how accurate this was in capturing our target patients, we used the T (primary tumor), N (regional lymph nodes), and M (distant metastasis) code variables available for 2004–2009 and found that the vast majority (97.5%) of individuals captured via our method had an M code indicating presence of distant metastasis. The remaining 2.5% of patients in whom distant metastasis was not present or could not be assessed had a T4 code (i.e., tumor invades beyond the Gerota fascia [including contiguous extension into the ipsilateral adrenal gland]). Additional details are available in Appendix 1 13. We excluded patients with nonclear cell cancer since the activity of most targeted agents in nonclear cell cancer is not well defined 14. Patients diagnosed at autopsy or by death certificate were also excluded (Fig. A1).

#### Comparison group

Since a comparison group of RCC patients who did not have access to targeted therapies was not available, we utilized a comparison group of adults aged ≥18 years who were newly diagnosed with prostate cancer (ICD‐O‐2/3 site codes C61.9) at an advanced or metastatic stage. Advanced prostate cancer patients were chosen as the comparison group for two key reasons. First, no significant innovation in treatments had taken place for this condition relative to RCC during the time frame of our study (see Appendix 2). Second, urologists manage both RCC and prostate cancer during the diagnostic stage, thus providing some level of control for trends in provider specialty characteristics. As with the study group, patients diagnosed at autopsy or by death certificate were excluded from the comparison group. Patients with diagnoses of both RCC and prostate cancer were excluded from both the comparison and the study groups.

### Outcome measures and covariates

We examined overall survival (OS) and cause‐specific survival (CSS) using two sets of outcomes: (1) survival time in months and (2) 1‐year and 3‐year survival rates. Survival was measured from the date of diagnosis of the advanced cancer. Outcomes were measured separately for patients with an advanced cancer diagnosis in the pretargeted therapy period versus targeted therapy period. In additional analyses, the targeted therapy period was further separated into individual years to allow examination of whether survival was better in 2010, when six targeted agents had become available, relative to 2006, when only two agents were available. For patients diagnosed in the pretargeted therapy era, survival outcome follow‐up ended on 31 December 2005 to ensure that any use of targeted therapies in later years did not contribute to improved survival. In addition to avoiding this bias in our pretargeted era survival estimates, this approach also leveled the playing field by ensuring comparable follow‐up periods for the pre‐ and postperiod groups, given that data for the patients diagnosed in the targeted therapy era was available only until 31 December 2010.

The main independent variable was an indicator of whether the patient was diagnosed with advanced cancer during the pretargeted therapy era or targeted therapy era. Control variables included age at diagnosis, sex, race, marital status, cancer grade (Appendix 3), and dummy variables for history of surgery on the primary site, history of radiation, and residence in states with laws requiring health plans to cover patient care costs in cancer clinical trials.

### Analysis

Sample characteristics were generated for both the study and comparison groups in the pre‐ and postperiods. Descriptive analyses and Kaplan–Meier survival curves compared unadjusted OS and CSS outcomes before and after the targeted therapy era among advanced RCC patients relative to advanced prostate cancer patients. Univariate and multivariate Cox regressions were estimated among advanced RCC patients and advanced prostate cancer patients to examine changes (pretargeted therapy era vs. targeted therapy era) in OS and CSS while controlling for differences in patient characteristics between the pretargeted and targeted therapy eras. Logistic regressions were used to examine changes in OS and CSS survival rates among advanced RCC patients and advanced prostate cancer patients. In sensitivity analyses, changes in OS and CSS survival rates were estimated using predicted survival changes and the standard error of those predictions based on Cox regression models. Differences in coefficients for the main independent variables between the advanced RCC and advanced prostate cancer groups were tested using a *z*‐statistic 15. Since patients diagnosed in 2004 and 2005 could have received new targeted therapies in clinical trials, we conducted a sensitivity analysis that excluded patients diagnosed in those years.

#### Subgroup analyses

We conducted several additional subgroup analyses. First, we repeated all analyses by nephrectomy status, since a majority of patients involved in clinical trials for the earlier targeted therapies had undergone nephrectomy. As a result, nephrectomy patients may have been more likely to be offered targeted therapies and thus have a better prognosis 5, 6, 7. Second, we repeated all analyses in subgroups of senior (age ≥ 65) and nonsenior (age < 65) patients to address the fact that the implementation of Medicare Part D in 2006 coincided with the beginning of our targeted therapy period and may have increased access to medications (and subsequently survival) for senior Medicare beneficiaries. Additionally, examination of the subgroup of nonsenior patients (very few of whom are likely to be Medicare eligible due to disability) provides results not impacted by this confounding Medicare policy change. Third, because all of the patients in our comparison group were male, we repeated analyses in subgroups for male and female advanced RCC patients and compared survival improvements in male RCC patients to prostate cancer patients.

## Results

A total of 13,670 patients initially diagnosed with advanced RCC and 25,990 patients initially diagnosed with advanced prostate cancer were included in the final study sample. Just over half of patients with each disease type were diagnosed in the pretargeted therapy era (Table 1).

**Table 1:** Sample characteristics for advanced renal cell carcinoma and advanced prostate cancer patients

	Advanced renal cell carcinoma (study group)			Advanced prostate cancer (control group)		
	Diagnosed in 2000–2005	Diagnosed in 2006–2010	*P*‐value	Diagnosed in 2000–2005	Diagnosed in 2006–2010	*P*‐value
Total (*N*)	7231	6439		13,924	12,066	
Age (mean, SD)	65.6 (12.8)	65.7 (12.8)	0.77	72.6 (11.1)	71.9 (11.6)	<0.01
Age group (%)						
18–54	1506 (20.8%)	1265 (19.6%)	0.01	879 (6.3%)	853 (7.1%)	<0.01
55–64	1885 (26.1%)	1830 (28.4%)		2522 (18.1%)	2630 (21.8%)	
65–74	1869 (25.8%)	1600 (24.8%)		3943 (28.3%)	3224 (26.7%)	
75 and up	1971 (27.3%)	1744 (27.1%)		6580 (47.3%)	5359 (44.4%)	
Gender (%)						
Female	2564 (35.5%)	2221 (34.5%)	0.02			
Male	4667 (64.5%)	4218 (65.5%)		13,924 (100.0%)	12,066 (100.0%)	
Race (%)						
White	6183 (85.5%)	5395 (83.8%)	<0.01	10,399 (74.7%)	9061 (75.1%)	0.04
Black	689 (9.5%)	594 (9.2%)		2630 (18.9%)	2153 (17.8%)	
Other	349 (4.8%)	438 (6.8%)		775 (5.6%)	747 (6.2%)	
Missing	10 (0.1%)	12 (0.2%)		120 (0.9%)	105 (0.9%)	
Marital status (%)						
Married	4201 (58.1%)	3632 (56.4%)	0.01	8117 (58.3%)	6766 (56.1%)	<0.01
Not married^a^	2795 (38.7%)	2544 (39.5%)		4961 (35.6%)	4507 (37.4%)	
Missing	235 (3.2%)	263 (4.1%)		846 (6.1%)	793 (6.6%)	
Cancer grade (%)						
1	136 (1.9%)	131 (2.0%)	<0.01	63 (0.5%)	25 (0.2%)	<0.01
2	709 (9.8%)	627 (9.7%)		2219 (15.9%)	498 (4.1%)	
3–4	1742 (24.1%)	1902 (29.5%)		7451 (53.5%)	7586 (62.9%)	
Cell type not determined	4644 (64.2%)	3779 (58.7%)		4191 (30.1%)	3957 (32.8%)	
Surgery on primary site (%)^b^						
Yes	2533 (35.0%)	2219 (34.5%)	0.72	1618 (11.6%)	1227 (10.2%)	<0.01
No	4654 (64.4%)	4184 (65.0%)		12,226 (87.8%)	10,773 (89.3%)	
Missing	44 (0.6%)	36 (0.6%)		80 (0.6%)	66 (0.5%)	
Nephrectomy or prostatectomy (%)						
Yes	2384 (33.0%)	2103 (32.7%)	0.70	200 (1.4%)	244 (2.0%)	<0.01
No	4847 (67.0%)	4336 (67.3%)		13,724 (98.6%)	11,822 (98.0%)	
Radiation (%)						
Yes	2160 (29.9%)	1733 (26.9%)	<0.01	3111 (22.3%)	2762 (22.9%)	<0.01
No	4986 (69.0%)	4667 (72.5%)		10,662 (76.6%)	9235 (76.5%)	
Missing	85 (1.2%)	39 (0.6%)		151 (1.1%)	69 (0.6%)	
Residence in state with cancer clinical trial laws^c^						
Yes	5527 (76.4%)	4934 (76.6%)	0.79	10,803 (77.6%)	9397 (77.9%)	0.57
No	1704 (23.6%)	1505 (23.4%)		3121 (22.4%)	2669 (22.1%)	

a Includes single, divorced, and widowed.

b Includes surgeries, local tumor excision or destruction, and laser ablation.

c Signifies laws requiring health plans to cover patient care costs in cancer clinical trials.

**Figure 1:**
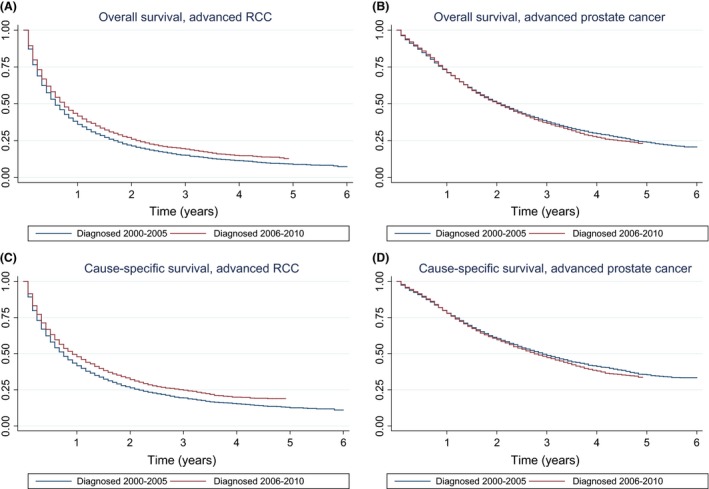
(A) Kaplan–Meier curves of overall survival (in years) for study group of advanced RCC patients diagnosed in 2000–2005 versus those diagnosed in 2006–2010. The difference in survival times between pretargeted therapy era and targeted therapy era was statistically significant (*P* < 0.01) based on Cox regression. (B) Kaplan–Meier curves of overall survival (in years) for control group of advanced prostate cancer patients diagnosed in 2000–2005 versus those diagnosed in 2006–2010. The difference in survival times between pretargeted therapy era and targeted therapy era was not statistically significant (*P* = 0.25) based on Cox regression. (C) Kaplan–Meier curves of cause‐specific survival (in years) for study group of advanced RCC patients diagnosed in 2000–2005 versus those diagnosed in 2006–2010. The difference in survival times between pretargeted therapy era and targeted therapy era was statistically significant (*P* < 0.01) based on Cox regression. (D) Kaplan–Meier curves of cause‐specific survival (in years) for control group of advanced prostate cancer patients diagnosed in 2000–2005 versus those diagnosed in 2006–2010. The difference in survival times between pretargeted therapy era and targeted therapy era was not statistically significant (*P* = 0.08) based on Cox regression.

Survival curves for each diagnostic group are shown in Figure 1A–D. Both OS and CSS were significantly higher in advanced RCC patients diagnosed in the targeted therapy era compared to those diagnosed in the pretargeted therapy era (Fig. 1A and C, respectively). No significant change was observed in the advanced prostate cancer group (Fig. 1B and D). Similarly, both median OS and CSS were 2 months greater in RCC patients diagnosed in the targeted therapy era compared to those diagnosed in the pretargeted therapy era (29% increase in OS: 7 vs. 9 months, *P *< 0.01; 22% increase in CSS: 9 vs. 11 months, *P *< 0.01); Table 2). Advanced prostate cancer patients did not show a statistically significant change in these measures.

**Table 2:** Overall and cause‐specific survival for advanced renal cell carcinoma and advanced prostate cancer patients

	Advanced renal cell carcinoma (study group)					Advanced prostate cancer (control group)				
	*N*	Diagnosed 2000–2005	Diagnosed 2006–2010	Change	*P*‐value	*N*	Diagnosed 2000–2005	Diagnosed 2006–2010	Change	*P*‐value
Median overall survival (months)										
All patients	13,670	7.0	9.0	+2.0	<0.01	25,990	25.0	24.0	−1.0	0.25
With nephrectomy	4487	16.0	22.0	+6.0	<0.01					
Without nephrectomy	9183	4.0	5.0	+1.0	<0.01					
Age < 65	6486	9.0	11.0	+2.0	<0.01	6884	31.0	31.0	0.0	0.73
Age ≥ 65	7184	6.0	7.0	+1.0	<0.01	19,106	23.0	22.0	−1.0	0.04
Male RCC	8885	7.0	9.0	+2.0	<0.01					
Female RCC	4785	7.0	8.0	+1.0	<0.01					
Median cause‐specific (months)										
All patients	13,670	9.0	11.0	+2.0	<0.01	25,990	35.0	33.0	−2.0	0.08
With nephrectomy	4487	18.0	26.0	+8.0	<0.01					
Without nephrectomy	9183	6.0	7.0	+1.0	<0.01					
Age < 65	6486	10.0	13.0	+3.0	<0.01	6884	38.0	36.0	−2.0	0.56
Age ≥ 65	7184	8.0	9.0	+1.0	<0.01	19,106	34.0	32.0	−2.0	0.03
Male RCC	8885	9.0	12.0	+3.0	<0.01					
Female RCC	4785	8.0	10.0	+2.0	<0.01					
1‐year survival (%)										
Overall survival										
All patients	12,274	32.8	38.0	+5.2	<0.01	22,022	69.1	69.8	+0.7	0.25
With nephrectomy	3856	58.1	67.0	+8.9	<0.01					
Without nephrectomy	8418	20.3	23.7	+3.4	<0.01					
Age < 65	5709	39.1	45.0	+5.9	<0.01	5644	80.8	80.8	+0.0	1.0
Age ≥ 65	6565	27.2	31.6	+4.4	<0.01	16,378	65.3	65.4	+0.1	0.84
Male RCC	7934	33.9	40.3	+6.4	<0.01					
Female RCC	4340	30.7	33.5	+2.8	0.06					
Cause‐specific										
All patients	12,274	43.0	48.5	+5.5	<0.01	22,022	78.2	78.3	+0.1	0.94
With nephrectomy	3856	62.2	70.7	+8.5	<0.01					
Without nephrectomy	8418	33.6	37.6	4.0	<0.01					
Age < 65	5709	45.5	52.2	+6.7	<0.01	5644	84.9	83.9	−1.0	0.35
Age ≥ 65	6565	40.8	45.2	+4.4	<0.01	16,378	76.1	76.0	−0.1	0.92
Male RCC	7934	44.0	50.3	+6.3	<0.01					
Female RCC	4340	41.2	45.0	+3.8	0.02					
3‐year survival (%)										
Overall survival										
All patients	11,076	11.9	16.5	+4.6	<0.01	17,360	36.2	35.4	−0.8	0.3
With nephrectomy	3088	26.5	36.4	+9.9	<0.01					
Without nephrectomy	7988	5.0	6.6	+1.6	0.02					
Age < 65	5019	15.7	20.8	+5.1	<0.01	4100	43.9	43.9	+0.0	0.97
Age ≥ 65	6057	8.8	12.7	+3.9	<0.01	13,260	33.8	32.4	−1.4	0.18
Male RCC	7091	12.5	16.8	+4.3	<0.01					
Female RCC	3985	10.9	15.8	+4.9	<0.01					
Cause‐specific										
All patients	12,274	24.3	30.1	+5.8	<0.01	22,022	53.5	52.5	−1.0	0.33
With nephrectomy	3856	33.5	43.5	+10.0	<0.01					
Without nephrectomy	8418	19.9	23.4	+3.5	0.01					
Age < 65	5709	24.1	30.6	+6.5	<0.01	5644	54.2	53.5	−0.7	0.71
Age ≥ 65	6565	24.5	29.6	+6.1	<0.01	16,378	53.2	52.1	−1.1	0.31
Male RCC	7934	24.8	30.1	+5.3	<0.01					
Female RCC	4340	23.5	29.9	+6.4	<0.01					

RCC, renal cell carcinoma.

**Figure A2:**
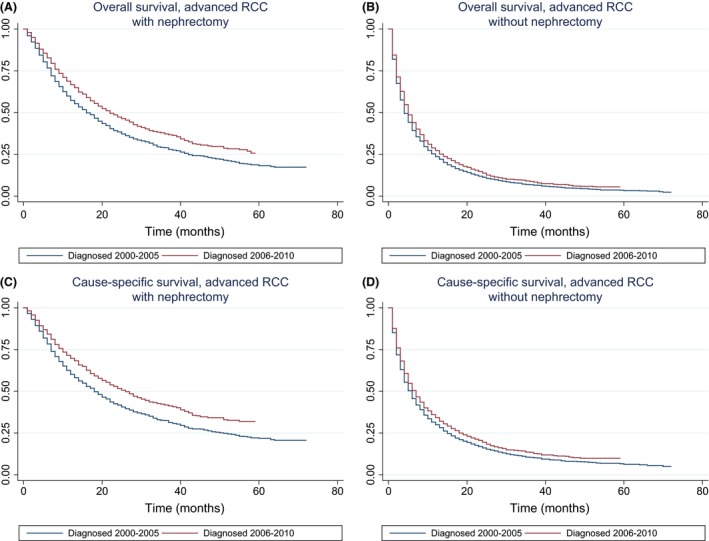
Kaplan–Meier curves of overall survival (in months) for advanced RCC patients (A) receiving and (B) not receiving nephrectomy, diagnosed in 2000–2005 versus those diagnosed in 2006–2010. The difference in survival times between pretargeted therapy era and targeted therapy era was statistically significant (*P* < 0.01) based on Cox regression, for both subsamples. Kaplan–Meier curves of cause‐specific survival (in years) for advanced RCC patients (C) receiving and (D) not receiving nephrectomy diagnosed in 2000–2005 versus those diagnosed in 2006–2010. *The difference in survival times between pretargeted therapy era and targeted therapy era was statistically significant (*P* < 0.01) based on Cox regression, in both subsamples.

In subgroup analyses (Table 2), median survival time among advanced RCC patients increased significantly in the targeted therapy era regardless of nephrectomy status, but the magnitude of this difference was greater for nephrectomy patients (6‐month and 8‐month increases in median OS and CSS, respectively, vs. 1 month for patients without nephrectomy, *P *< 0.01) (Table 2 and Fig. A2). A similar pre–post pattern was observed for age, with patients less than 65 years showing a greater median increase in OS and CSS. Subgroup analysis by gender indicated larger increases for male RCC patients relative to female RCC patients (2 months vs. 1 month for OS, 3 months vs. 2 months for CSS). Similar patterns were observed for 1‐year and 3‐year OS and CSS survival rates.

As shown in Table 3, advanced RCC patients diagnosed in the targeted therapy era had a significantly lower hazard of death (i.e., longer survival time) compared to those diagnosed in the pretargeted therapy era (OS hazard ratio [HR] 0.86, 95% CI 0.82–0.90, *P *< 0.01; CSS HR 0.84, 95% CI 0.80–0.88, *P* < 0.01). In contrast, pre–post change was smaller among advanced prostate cancer patients (OS HR 0.97, 95% CI 0.93–1.00, *P* = 0.08; CSS HR 0.96, 95% CI 0.92–1.00, *P *= 0.04). *z*‐statistics indicated that advanced RCC patients had significantly larger improvements in survival time compared to advanced prostate cancer patients (OS *z* = 4.31, *P *< 0.01; CSS *z* = 4.27, *P* < 0.01). Subgroup analyses across nephrectomy status, senior age status, and gender demonstrated similar results. Sensitivity analyses excluding patients diagnosed in 2004 and 2005 were consistent with these results (Table A1).

**Table 3:** Overall and cause‐specific survival for advanced RCC and prostate cancer patients diagnosed 2006–2010 versus 2000–2005.^a^

	*N*	Overall survival			Cause‐specific survival		
		Hazard ratio	95% CI	*P*‐value	Hazard ratio	95% CI	*P*‐value
Overall sample							
RCC	13,670	0.86	0.82–0.90	<0.01	0.84	0.80–0.88	<0.01
Prostate	25,990	0.97	0.93–1.00	0.08	0.96	0.92–1.00	0.04
*z*‐statistic^b^		4.31	na	<0.01	4.27	na	<0.01
RCC with nephrectomy	4487	0.76	0.70–0.82	<0.01	0.73	0.67–0.80	<0.01
Prostate	25,990	0.97	0.93–1.00	0.08	0.96	0.92–1.00	0.04
*z*‐statistic		5.42	na	<0.01	3.11	na	<0.01
RCC without nephrectomy	9183	0.90	0.86–0.94	<0.01	0.88	0.84–0.93	<0.01
Prostate	25,990	0.97	0.93–1.00	0.08	0.96	0.92–1.00	0.04
*z*‐statistic		2.41	na	0.01	2.33	na	0.02
RCC male	8885	0.84	0.79–0.88	<0.01	0.82	0.78–0.87	<0.01
Prostate	25,990	0.97	0.93–1.00	0.08	0.96	0.92–1.00	0.04
*z*‐statistic		4.49	na	<0.01	4.22	na	<0.01
RCC female	4785	0.89	0.83–0.96	<0.01	0.86	0.80–0.93	<0.01
Prostate	25,990	0.97	0.93–1.00	0.08	0.96	0.92–1.00	0.04
*z*‐statistic		1.97	na	0.03	2.40	na	0.02
Age < 65							
RCC	6486	0.83	0.78–0.88	<0.01	0.81	0.75–0.86	<0.01
Prostate	6884	0.90	0.83–0.97	<0.01	0.90	0.83–0.97	0.01
*z*‐statistic		1.59	na	0.19	1.99	na	0.09
Age ≥ 65							
RCC	7184	0.88	0.83–0.94	<0.01	0.87	0.82–0.93	<0.01
Prostate	19,106	0.99	0.95–1.03	0.58	0.98	0.93–1.03	0.40
*z*‐statistic		3.14	na	<0.01	2.92	na	<0.01

RCC, renal cell carcinoma; CI, confidence interval.

a Hazard ratios for patients diagnosed in 2006–2010 relative to the reference category of patients diagnosed in 2000–2005 based on Cox regressions adjusting for age at diagnosis, sex, race, marital status, cancer grade, and dummy variables for having surgery on primary site, having radiation, and residence in states with laws requiring health plans to cover patient care costs in cancer clinical trials.

b *z*‐statistic: (b1 – b2)/sqrt(se(b1)^2^ + se(b2)^2^), where b1 is the coefficient for RCC, b2 is the coefficient for prostate, se(b1) is the standard error for b1, and se(b2) is the standard error for b2.

Finally, logistic regression results showed that RCC patients diagnosed in the targeted therapy era had higher odds of 1‐ and 3‐year survival compared to patients diagnosed in the pretargeted therapy era. Improvement in survival odds was significantly larger among patients with advanced RCC than patients with advanced prostate cancer (Table 4). Sensitivity analyses for 1‐ and 3‐year survival rates based on Cox regression models showed consistent findings (Table A2). As shown in Table 5, more detailed year‐to‐year analysis revealed that compared to patients diagnosed with advanced RCC before 2006, hazard ratios for posttargeted therapy era patients dropped nearly every year (from 0.89 in 2006 to 0.82 in 2009 and 0.79 in 2010 for OS and from 0.89 in 2006 to 0.81 in 2009 and 0.74 in 2010 for CSS); HRs in 2010 were significantly lower than HRs in 2006.

**Table 4:** One‐ and 3‐year survival for advanced RCC and advanced prostate cancer patients diagnosed 2006–2010 versus 2000–2005.^a^

	*N*	Overall survival			Cause‐specific survival		
		Odds ratio	95% CI	*P*‐value	Odds ratio	95% CI	*P*‐value
1‐year survival							
RCC	12,274	1.35	1.23–1.47	<0.01	1.29	1.19–1.40	<0.01
Prostate	22,022	1.08	1.01–1.15	0.02	1.06	0.99–1.13	0.11
*z*‐statistic^b^		4.08	na	<0.01	3.70	na	<0.01
3‐year survival							
RCC	11,076	1.56	1.33–1.83	<0.01	1.36	1.21–1.53	<0.01
Prostate	17,360	1.13	1.04–1.23	<0.01	1.11	1.03–1.20	<0.01
*z*‐statistic		3.51	na	<0.01	2.80	na	0.01

RCC, renal cell carcinoma; CI, confidence interval.

a Odds ratios for patients diagnosed in 2006–2010 relative to the reference category of patients diagnosed in 2000–2005 based on logistic regressions adjusting for age at the first diagnosis, sex, race, marital status, cancer grade, and dummy variables for having surgery on primary site, having radiation, and residence in states with laws requiring health plans to cover patient care costs in cancer clinical trials.

b *z*‐statistic: (b1 – b2)/sqrt(se(b1)^2^ + se(b2)^2^), where b1 is the coefficient for RCC, b2 is the coefficient for prostate, se(b1) is the standard error for b1, and se(b2) is the standard error for b2.

**Table 5:** Overall and cause‐specific survival for advanced RCC patients diagnosed 2006–2010 versus 2000–2005.^a^

Year of diagnosis	Overall survival (*N* = 13,670)			Cause‐specific survival (*N* = 13,670)		
	Hazard ratio	95% CI	*P*‐value	Hazard ratio	95% CI	*P*‐value
2006^b^	0.89	0.83–0.96	0.001	0.89	0.82–0.96	0.002
2007	0.90	0.84–0.96	0.003	0.87	0.81–0.94	<0.001
2008	0.84	0.78–0.90	<0.001	0.82	0.76–0.89	<0.001
2009	0.82	0.76–0.89	<0.001	0.81	0.75–0.88	<0.001
2010^b^	0.79	0.71–0.88	<0.001	0.74	0.66–0.83	<0.001

RCC, renal cell carcinoma; CI, confidence interval.

a Hazard ratios for patients diagnosed in 2006–2010 relative to the reference category of patients diagnosed in 2000–2005 based on Cox regressions adjusting for age at diagnosis, sex, race, marital status, cancer grade, and dummy variables for having surgery on primary site, having radiation, and residence in states with laws requiring health plans to cover patient care costs in cancer clinical trials.

b Chi‐square test showed that the hazard ratio for 2010 is significantly lower than the hazard ratio for 2006 (*P *= 0.043 for overall survival and *P* = 0.007 for cause‐specific survival).

## Discussion

This study finds that advanced renal cell carcinoma patients in the United States diagnosed in the targeted therapy era (2006–2010) had a significantly longer survival time and higher 1‐ and 3‐year survival rates compared to patients diagnosed in the pretargeted therapy era (2000–2005). Furthermore, overall and cause‐specific survival was higher in the last year (2010) of our targeted therapy period, by which time six novel targeted therapies had become available, compared to the first year (2006) when only two targeted agents were available. Our results help to characterize the potential benefits of multiple innovations in the management of advanced RCC. The more pronounced differences at the end of our postperiod may also reflect the fact that new treatments may take time to be adopted more broadly.

Since population‐based improvements in survival could be due to a variety of factors, we used advanced prostate cancer patients, for whom comparable treatment innovations were not available during the study period, as a comparison group to help control for whether survival advantages seen in advanced RCC patients may have been due to factors other than RCC‐specific care. Patients with advanced prostate cancer had minimal improvement in survival time or survival rates during the same time interval. These findings serve as a validation of the results reported in previous pre–post only studies, which may otherwise be viewed with skepticism due to the lack of a contemporaneous control group 8, 10. Furthermore, our estimated improvements in survival are greater in magnitude than those reported in previous studies, most likely because our study design addressed methodological limitations of those studies. Our findings are also consistent with a recent meta‐analysis based on clinical trials data showing an increase in overall survival associated with targeted therapy use 16.

Nevertheless, several caveats deserve mention. First, the SEER database does not provide any information on systemic therapy, so our analysis was limited by examining survival among all advanced RCC patients regardless of whether they received treatment. Since this means any survival benefit from targeted therapies would be spread across patients who received these targeted therapies and those who did not, our results (median increase of 2 months among *all* advanced RCC patients in the U.S.) are likely an underestimate of the survival impact in treated patients. In keeping with this idea, a population‐based study using national data from Denmark found that the proportion of advanced RCC patients receiving treatment increased from 64% to 75% between 2006 and 2010; those receiving targeted therapy as first‐line treatment increased from 22% to 75%; and these increases corresponded with an increase in median overall survival of 5.7 months (11.5 vs. 17.2 months*, P* = 0.04) in treated patients, while median overall survival for untreated patients remained stable at 3.0 months 17.

There is limited analogous data in the published literature regarding what proportion of advanced RCC patients in the U.S. are receiving targeted therapies. One study using the U.S. National Cancer Database Public Benchmark Reports indicated that 23% of patients from 2006 to 2008 with advanced RCC at initial diagnosis did not receive anti‐cancer therapy 18, yet no details were provided on the type of cancer therapy received by the remaining 77% of the patients in this database. The fact that cancer treatment in Denmark is free suggests that our estimates of increases in survival among all patients with advanced RCC, by comparison, are likely weighted downward since a larger proportion of the patients in our targeted therapy era sample may not have had access to these therapies due to variations in insurance availability and high patient out‐of‐pocket costs in the U.S. 17. Of note, patients who had undergone nephrectomy showed a greater median increase in overall survival across the pre‐ and postperiods than those patients who did not (6 months vs. 1 month). Nephrectomy status may have been a proxy for patients receiving targeted therapies in our data, as patients who are good surgical candidates are likely healthier than the general patient population of advanced RCC patients and more likely to be treated with systemic therapy 19. Therefore, the nephrectomy group may better represent the benefit of targeted therapies compared to the nonnephrectomy group.

A second important caveat is that the SEER data capture cancer stage at the time of initial diagnosis only. As a result, our sample does not include patients with localized disease who underwent nephrectomy with curative intent and then later developed metastatic disease. Such patients, who may have lower volume disease than patients who present with high volume, symptomatic metastatic disease at the time of diagnosis, may have better outcomes when receiving targeted therapy 20. Thus, our study findings may be a conservative estimate of the survival improvements seen in patients whose RCC progresses to an advanced stage after initial treatment.

Third, the SEER data, and other secondary databases, lack information on clinical trial enrollment. If patients in the pretargeted therapy era had clinical trial access to the targeted agents before they entered the market, then the difference in survival between the pretargeted and targeted therapy eras would be dampened. Any such effect is likely to be small, however, as it is estimated that less than 5% of U.S. adult cancer patients participate in clinical trials 21. Also, we included a covariate on whether the patient resided in states with laws requiring that insurers cover costs in cancer clinical trials as a proxy for patients who may have had better access to enrollment in clinical trials.

In addition to the possibility of underestimation of the true impact of targeted therapies, some limitations in our data may have led to an overestimate of effects. Although we controlled for differences between patients in the pre‐ and postperiods on relevant clinical and sociodemographic factors such as age and cancer grade, the SEER database does not contain other information (such as laboratory values, Memorial Sloan‐Kettering Cancer Center risk score) that could impact outcomes. In addition, advanced RCC patients diagnosed in the targeted period might have had lower volume disease than patients diagnosed in the pretargeted period as a result of earlier detection due to improvements in diagnostic imaging. If this is the case, the improvement in survival observed among RCC patients in our study might be reflective of patients presenting with lower volume or indolent disease (rather than access to targeted therapies) during the targeted period compared to the pretargeted period. That said, it is notable that the percentage of patients receiving cytoreductive nephrectomy, who likely represent a group of patients with lower volume and more indolent disease 19, 22, was stable across the pretargeted and targeted therapy era. Similarly, mean age of patients at initial diagnosis, which could be another marker of earlier identification of patients with lower volume disease, also remained stable (65.6 years pretargeted vs. 65.7 years posttargeted, *P* = 0.77). Furthermore, while we tried to control for other contemporaneous trends in diagnostic, clinical, and palliative care for advanced cancers in general by including advanced prostate cancer patients as the control group, it is possible that prostate cancer patients may not have been affected by contemporaneous trends in exactly the same manner as the advanced RCC patients. In addition, the population‐based improvements in survival observed in our study could still be due to other improvements in RCC‐specific care over time rather than just the introduction of targeted therapies.

Finally, we had to rely on the only variable that is consistently available in each year of our study time frame to capture our sample of metastatic RCC patients. On the basis of our validation work on the variable, we found that we may have captured a very small percentage (2.5% in 2004 to 2009 data) of patients with T4 stage but not metastatic disease who would not have been candidates for targeted therapy. However, given that we used the same approach to capture pre‐ and postperiod patients and only a very small percentage might be nonmetastatic, the impact of this limitation on our results is expected to be minimal.

## Conclusion

Patients first diagnosed with advanced RCC during the targeted therapy era (2006–2010) had better survival outcomes than advanced RCC patients diagnosed during the pretargeted therapy era (2000–2005). Furthermore, survival was better in 2010, when six targeted agents were available, relative to 2006, when only two agents were on the market. Future studies should directly examine the survival improvements associated with targeted therapies in the real‐world setting.

## Conflict of Interest

Dr. Subedi and Ms. Davis report employment with Pfizer and owning Pfizer stock. Dr. Doshi has served as a consultant and/or member of an advisory board for Alkermes, Boehringer Ingelheim, Forest, Merck, and Shire, receiving honoraria; had grants or has pending grants from Amgen, Humana, Merck, Pfizer, PhRMA, and the National Pharmaceutical Council; and has a spouse who holds stocks in Merck and Pfizer. Dr. Wong reports funding from Pfizer and Tokai and support for travel from Tokai. Drs. Li, Haas, and Armstrong do not have any conflicts of interest to report.

[Correction added on 05 January 2016, after first online publication: The Conflict of Interest was previously omitted and has now been added in this current version.]

## Appendix 1

SEER cancer stage variable (hist_ssg_2000).

**Table:**

0	In situ
1	Localized only
2	Regional by direct extension only
3	Regional lymph nodes involved only
4	Regional by both direct extension and lymph
5	Regional, NOS
7	Distant site(s)/node(s) involved
9	Unknown/unstaged/unspecified/DCO

As per our confirmation with SEER staff, this is the only variable that is consistently available in each year of our study time frame (2000–2010). The T (primary tumor), N (regional lymph nodes) and M (distant metastasis) code variables are available only for the years 2004–2009, which if used for sample selection do not permit sufficient follow‐up for an assessment of survival rates in the pretargeted therapy era (2000–2005).

Nevertheless, we have cross‐checked the SEER summary stage variable against the TNM variables using 2004–2009 data wherein both types of variables are available. Validation work showed that the vast majority (97.5%) of the 7411 patients selected by our summary stage variable requiring distant site(s)/node(s) involved during this time frame includes patients who have an M code indicating presence of distant metastasis. The remaining 2.5% of patients in whom distant metastasis was not present or could not be assessed had a T4 code (i.e., tumor invades beyond the Gerota fascia [including contiguous extension into the ipsilateral adrenal gland]). In summary, our sample selected over 2000–2010 using the SEER summary stage variable with distant site(s)/node(s) involved primarily includes stage IV RCC patients.

## Appendix 2

Docetaxel chemotherapy was approved for prostate cancer in 2004; 23, 24 Sipuleucel‐T and Cabazitaxel were approved at the end of our study period (mid‐2010) 25, 26. However, the Centers for Medicare and Medicaid Services (CMS) did not issue a coverage decision for Sipuleucel‐T until November 2010, so its use was likely limited. Cabazitaxel is indicated after docetaxel failure and can be associated with toxicities, so its use was also likely limited. Abiraterone and enzalutamide were not yet available for this population as well. Thus, there were no major changes in treatment that would likely contribute to significant changes in survival rates among advanced prostate cancer patients during 2000–2010.

## Appendix 3

### SEER cancer grade variable

Grade I; grade i; grade 1; well differentiated; differentiated, NOS.

Grade II; grade ii; grade 2; moderately differentiated; intermediate differentiation.

Grade III; grade iii; grade 3; poorly differentiated; differentiated.

Grade IV; grade iv; grade 4; undifferentiated; anaplastic.

**Table A1:** Overall and cause‐specific survival for advanced RCC and prostate cancer patients diagnosed in 2006–2010 versus those diagnosed in 2000–2003.^a^

	Overall survival			Cause‐specific survival		
	Hazard ratio	95% CI	*P*‐value	Hazard ratio	95% CI	*P*‐value
RCC (*N* = 11,336)	0.82	0.79–0.86	<0.001	0.80	0.76–0.84	<0.001
Prostate (*N* = 21,325)	0.95	0.91–0.99	0.006	0.94	0.90–0.98	0.003
*z*‐statistic^b^	5.41	na	<0.001	5.33	na	<0.001

RCC, renal cell carcinoma; CI, confidence interval.

a Hazard ratios for patients diagnosed in 2006–2010 relative to the reference category of patients diagnosed in 2000–2003 based on Cox regressions adjusting for age at diagnosis, sex, race, marital status, cancer grade, and dummy variables for having surgery on primary site, having radiation, and residence in states with laws requiring health plans to cover patient care costs in cancer clinical trials.

b *z*‐statistic: (b1 – b2)/sqrt(se(b1)^2^ + se(b2)^2^), where b1 is the coefficient for RCC, b2 is the coefficient for prostate, se(b1) is the standard error for b1, and se(b2) is the standard error for b2.

**Table A2:** Changes in overall and cause‐specific 1‐year and 3‐year survival for advanced RCC and prostate cancer patients diagnosed 2006–2010 versus 2000–2005.^a^

	Overall survival		Cause‐specific survival	
1‐year survival rate change (after–before)				
	Estimate	Std. err.	Estimate	Std. err.
RCC (*N* = 13,670)	0.045	0.006	0.052	0.007
Prostate cancer (*N* = 25,990)	0.007	0.003	0.006	0.003
	*z*‐statistic^b^	*P*‐value	*z*‐statistic^b^	*P*‐value
	5.38	<0.01	6.22	<0.01
3‐year survival rate change (after–before)				
	Estimate	Std. err.	Estimate	Std. err.
RCC (*N* = 13,670)	0.037	0.005	0.048	0.006
Prostate cancer (*N* = 25,990)	0.011	0.006	0.012	0.006
	*z*‐statistic^b^	*P*‐value	*z*‐statistic^b^	*P*‐value
	3.42	<0.01	4.20	<0.01

RCC, renal cell carcinoma.

a Based on Cox regressions adjusting for age at diagnosis, sex, race, marital status, cancer grade, and dummy variables for having surgery on primary site, having radiation, and residence in states with laws requiring health plans to cover patient care costs in cancer clinical trials.

b *z*‐statistic: (b1 – b2)/sqrt(se(b1)^2^ + se(b2)^2^), where b1 is the coefficient for RCC, b2 is the coefficient for prostate, se(b1) is the standard error for b1, and se(b2) is the standard error for b2.
